# Assessing Approaches of Human Inhalation Exposure to Polycyclic Aromatic Hydrocarbons: A Review

**DOI:** 10.3390/ijerph18063124

**Published:** 2021-03-18

**Authors:** Xuan Zhang, Lu Yang, Hao Zhang, Wanli Xing, Yan Wang, Pengchu Bai, Lulu Zhang, Kazuichi Hayakawa, Akira Toriba, Yongjie Wei, Ning Tang

**Affiliations:** 1 Graduate School of Medical Sciences, Kanazawa University, Kakuma-machi, Kanazawa 920-1192, Japan; zhangxuan@stu.kanazawa-u.ac.jp (X.Z.); veronicayl@stu.kanazawa-u.ac.jp (L.Y.); zhanghao@stu.kanazawa-u.ac.jp (H.Z.); xingwanli@stu.kanazawa-u.ac.jp (W.X.); wangyan@stu.kanazawa-u.ac.jp (Y.W.); baipengchu@stu.kanazawa-u.ac.jp (P.B.); 2 Institute of Nature and Environmental Technology, Kanazawa University, Kakuma-machi, Kanazawa 920-1192, Japan; zhang-lulu@se.kanazawa-u.ac.jp (L.Z.); hayakawa@p.kanazawa-u.ac.jp (K.H.); 3 School of Pharmaceutical Sciences, Nagasaki University, Bunkyo-machi, Nagasaki 852-8521, Japan; toriba@nagasaki-u.ac.jp; 4 State Key Laboratory of Environmental Criteria and Risk Assessment, Chinese Research Academy of Environment Sciences, Beijing 100012, China; 5 Institute of Medical, Pharmaceutical and Health Sciences, Kanazawa University, Kakuma-machi, Kanazawa 920-1192, Japan

**Keywords:** polycyclic aromatic hydrocarbon, human exposure, time-activity patterns, modeling

## Abstract

Polycyclic aromatic hydrocarbons (PAHs) are a class of important organic pollutants widely emitted from anthropogenic activities, with a general distribution in the gas and particulate phases. Some PAHs are carcinogenic, teratogenic, and mutagenic. Inhalation exposure to PAHs is correlated with adverse health outcomes in the respiratory and cardiovascular systems. Thus, it is significant to determine the exposure level of the general population. This study summarizes the evaluation methods for PAH exposure, focusing on different exposure parameters. External exposure can be determined via the collection of the environmental pollution concentration through active samplers or passive samplers during environmental monitoring or personal sampling. Time-activity patterns give critical exposure information that captures the exposure period, origin, and behaviors. Modeling is a labor-less approach for human exposure estimation, and microenvironmental exposure requires specific research. It is important to select appropriate methods to quantify the exposure level to provide accurate data to establish the exposure–risk relationship and make scientific suggestions for the protection of public health.

## 1. Introduction

Polycyclic aromatic hydrocarbons (PAHs) are a large class of persistent organic compounds (over 100 species) composed of two or more fused aromatic rings. PAHs are generated from the incomplete combustion of organic materials [1,2,3,4,5,6], which widely involve natural sources, such as volcano eruptions and forest fires, and anthropogenic activities as a dominant contributor, such as factory emissions, vehicle exhaust, residential heating, and environmental tobacco smoking [7,8,9,10,11].

PAHs have attracted great attention due to their adverse effects on human health. Some species are characterized with carcinogenicity, teratogenicity, and genotoxicity [12]. The International Agency for Research on Cancer Monographs Program has listed the carcinogenicity of 60 individual PAHs [13]. Among them, benzo[*a*]pyrene (BaP) is classified in Group 1 as a carcinogen to humans. Cyclopenta[*c,d*]pyrene (CPP), dibenz[*a,h*]anthracene (DBA), and dibenzo[*a,l*]pyrene (DlP) are listed in Group 2A with probable carcinogenicity to humans. Benz[*j*]aceanthrylene, benz[*a*]anthracene (BaA), benzo[*b*]fluoranthene (BbF), benzo[*j*]fluoranthene (BjF), benzo[*k*]fluoranthene (BkF), benzo[*c*]phenanthrene, chrysene (Chr), dibenzo[*a,h*]pyrene (DhP), dibenzo[*a,i*]pyrene (DiP), indeno[1,2,3-*cd*]pyrene (IDP), and 5-methylchrysene (5MC) are classified as possibly carcinogenic to humans (Group 2B). On the other hand, short/long-term PAH exposure can induce and strengthen respiratory symptoms and airway illnesses such as asthma, chronic obstructive pulmonary diseases, and even cancers [14,15,16,17]. PAH exposure is also correlated with reproductive dysfunction [18,19,20]. Prenatal exposure to PAHs could lead to several birth defects [21,22,23]. The U.S. Environmental Protection Agency listed 16 PAHs as the primary control pollutants, including naphthalene (Nap), acenaphthylene (Acy), acenaphthene (Ace), fluorene (Fle), phenanthrene (Phe), anthracene (Ant), fluoranthene (FR), pyrene (Pyr), BaA, Chr, BbF, BkF, BaP, DBA, benzo[*ghi*]perylene (BghiP) and IDP [24]. The European Union classified 15 + 1 PAH species as priorities in food, involving benzo[*c*]fluorene (BcF), BaA, CPP, Chr, 5MC, BbF, BjF, BkF, BaP, BghiP, DBA, IDP, dibenzo[*a,e*]pyrene (DeP), DhP, DiP, and DlP [25]. Moreover, due to a strong carcinogenicity, an environmental standard was set for the ambient level of BaP in some countries, such as a 24-h standard of 2.5 ng/m^3^ and an annual standard of 1.0 ng/m^3^ in China [26] and an annual level of 1.0 μg/m^3^ as a target value in Europe [27].

PAHs are ubiquitously distributed in the particulate and gaseous phases in the atmosphere, defined by their physiochemical properties, and impacted by meteorological conditions. Generally, low molecular weight PAHs (LMW-PAHs) with 2–3 rings are abundant in the gaseous phase. Four-ring PAHs with middle molecular weights (MMW-PAHs) are distributed in both phases with the influence of meteorological parameters. Five- and six-ring PAHs (HMW-PAHs) with high molecular weights are prone to exist in the particulate phase. Furthermore, particulate PAHs show different size distributions in particulate matter (PM); that is, freshly emitted PAHs are prone to distribute in finer size modes than those in ambient air [28]. In addition, PAHs can have a negative effect not only on local sites but also on downwind sites during long-range transport due to their long atmospheric lifetime [29,30,31,32,33,34]. The heterogeneous distribution and human health risk of PAHs indicates the importance of exploring the bodily burden and establishing an exposure–risk relationship.

Humans are exposed to PAHs through inhalation and oral and dermal pathways. According to the World Health Organization (WHO) [35], air pollution exposure was related to 4.2 million deaths in 2016, indicating an urgency for human exposure evaluation to air pollutants. The daily intake volume of air is 3.6 to 16.3 m^3^ for different age groups [36], suggesting a continuous and stable air pollution exposure of the public via inhalation. On the other hand, the oral route is related to life habits such as eating contaminated food. Dermal exposure also results from contaminated air while it is restricted by effective contact area and infiltration rate. Furthermore, inhalation exposure can directly pose an adverse effect on human respiratory system, which requires an accurate assessment.

Generally, the impact of environmental factors on human health is quantitatively evaluated by an exposure–response function in epidemiological studies. This function includes three key points: an accurate exposure dose, a clear health outcome intensity, and a scientific statistical analysis. The induced health effect is determined by the exposure length, route, PAH concentration, and toxicity, which should be clarified to evaluate its biological efficiency [37]. The current research has summarized and evaluated the most commonly used PAH exposure evaluation methods via inhalation based on their principles, advantages and disadvantages.

## 2. Evaluation Method

**Figure 1:**
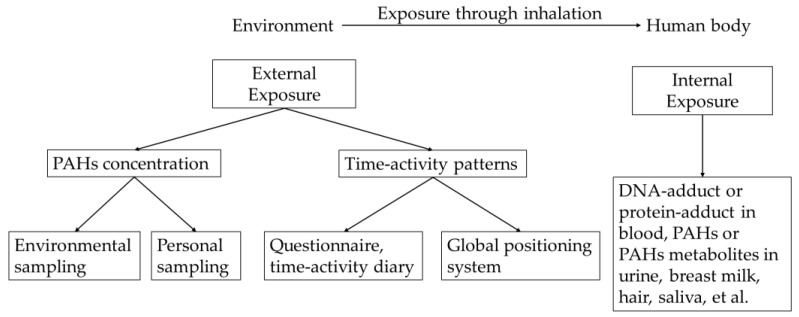
Flow chart of evaluation methods for polycyclic aromatic hydrocarbons (PAHs) exposure.

According to the study objectives, appropriate measuring methods should be selected and designed to reflect the actual exposure status of targeted individuals and a larger population within a reasonable range. In exposure evaluation studies, two kinds of exposures are typically characterized and measured (Figure 1), which are external and internal exposure according to the exposure stage.

External exposure (Figure 1) is the air pollution concentration level of the environment where people dwell for a period, and can describe the exposure situation of individuals or populations. The exposure level can be directly estimated or predicted by modeling work based on pollution-related data and human time-activity patterns. Pollution-related data include the concentration of PAHs and impacting factors such as the meteorological index [38]. Basically, external exposure can be obtained by environmental sampling (conducted with fixed monitors) and individual-based measuring methods (conducted by personal mobile monitors) or a combination with active or passive samplers. In addition, the exposure period and behaviors are generally obtained by questionnaire and time-activity diary. The daily time-location patterns of subjects can also be tracked by a global positioning system.

The exposure level (C_i_) in a specific location is the result of the measured level multiplied by the respective dwelling time (t_i_). The total exposure level (C) is the sum of a series of microenvironmental exposures as follows (Equation (1)):

C = ∑C_i_ × t_i_ (1)

The average daily dose (ADD) by inhalation during the short- and long-term exposure can be estimated by combining a series of exposure parameters as in Equation (2) [39], in which IR is the inspiration rate, m^3^/d or kg/d; ET is the exposure time, h/d; EF is the exposure frequency, d/a; ED is the exposure duration, a; BW is the body weight; and AT is the average exposure time. In addition to calculating the sum of the microenvironmental exposure, the respiratory exposure can be further refined into the exposure dose during different activities because the difference in the intensity of the activity will cause the difference in the respiratory volume [40] (Equation (2)).

ADD = C × IR × ET × EF × ED/BW/AT(2)

Internal exposure (Figure 1) indicates the body-absorbed PAH dose and is closer to the biologically effective content. After being inhaled, some amounts of PAHs are excluded through behaviors of the respiratory system such as coughing and sneezing. The remaining PAHs can further enter the lung and other organs and go through the metabolism. The internal exposure of PAHs is commonly represented by the parent PAH or metabolite content in human body specimens such as blood [41,42,43,44,45], urine [46,47,48,49,50,51], hair [52,53,54], breast milk [55,56,57], and saliva [58,59,60]. Nevertheless, it should be noted that internal exposure is a reflection of all exposure pathways. It is hard to extract the contribution of each route [47] to trace the exposure source and behavior and further provide suggestions to target control measures. On the other hand, the baseline of internal PAHs exposure levels in the human body is critically impacted by individual lifestyles and metabolite capacity [43,50], which makes it difficult to clarify the proportion of inhalation exposure.

Thus, in this study, external exposure will be dominantly discussed, involving the methods to collect air pollutant concentration and other exposure-related data.

### 2.1. Pollution-Related Data Based on Environmental Sampling

Environmental sampling is conducted through one or several stationary samplers set in the surrounding environment of the target population during sampling periods.

Table 1 listed previous studies of PAHs exposure measured by stationary sampling. Ambient PAH levels can reflect the regional outdoor atmospheric conditions, which is the traditional data source used to evaluate the exposure level of the surrounding population. Pei et al. [61] collected the short-term exposure to PAH-bound particulate matter (PM_2.5_) of 175 elderly adults by samplers set in 5 communities in Beijing; the PAH level exhibited mean values of 44.2 and 47.1 ng/m^3^ during two sampling periods. Wu et al. [62] measured PAH concentrations ranging from 0.8 to 18.3 mg kg^−1^ in the street dust obtained from bus stops to assess the exposure level of daily commuters. Ramírez et al. [63] monitored the concentration of 18 PAHs in particulate and gas phase in 3 nearby industry sites and found that the inhalation cancer risk of PAHs exposure for surrounding dwellers was 1.2 × 10^−4^, which was 10 times higher than the guidelines suggested by the WHO.

The indoor air environment also has many sources, e.g., from cooking, cleaning, and domestic heating, which significantly contribute to human exposure [64]. Du et al. [65] detected elevated levels of 28 PAHs in gaseous and particulate phases in the kitchen in rural households located in northern (3132 ng/m^3^) China, compared to southern China (594 ng/m^3^). In Xi’an, a northwestern city in China, He et al. [66] found a higher PAH concentration in the rural household conducting cooking by mixed-fuel than those using coal, which was 116 and 103 ng/m^3^, respectively. In Chen et al. [46], a high outdoor concentration level of 16 PAHs (77.7 ng/m^3^) was detected in a county near a coal chemistry factory, while a concentration level of 1.17 ng/m^3^ was detected in an agricultural county. However, the indoor level was the reverse. Zhu et al. [67] measured the indoor and outdoor PAHs concentration of an office in Jinan, China and found that office workers were 10 times more likely to be at risk of cancer in autumn than summer in both indoor and outdoor environments. In our previous studies, the exposure level of children in school environments in Beijing and Shanghai was investigated. The exposure level of 11 PAHs during the heating period was approximately 25 times higher than that during the non-heating period both indoors and outdoors in Beijing [68]. Levels of 9 PAHs were found to be approximately 4 times higher in the cold season than in the warm season at two urban and suburban elementary schools in Shanghai with no domestic heating activity [69]. Similarly, Chen et al. [70] measured higher PAH levels at an outdoor site (61.2 ng/m^3^) than its surrounding three indoor environments (34.1 ng/m^3^ at a students’ dormitory, 32.1 ng/m^3^ at a residential home and 39.8 ng/m^3^ at an office) in urban Beijing. PAH concentration in ambient air was also elevated compared to the concurrent residential indoor levels in Hong Kong, which were 3.9 and 3.0 ng/m^3^, respectively [71].

Individuals have relatively stable time-location-activity patterns, which makes it reasonable to measure and combine microenvironmental exposures to estimate personal exposure in a statistical model. Place-based measurements can generate an exhaustive description of the specified environmental exposure to clarify the key sources, crucial PAH species, and human exposure behaviors, which is beneficial for providing scientific suggestions for standard formation. Moreover, this approach could lower the sampling burden of the subjects.

### 2.2. Pollution-Related Data Based on Personal Sampling

Personal sampling is participant-based; i.e., portable samplers with high spatial resolution are carried by the subjects during the sampling periods to describe individual variations rather than environmental variations in the exposure level. Portable instruments are commonly packed in a sampling box or backpack or pinned to the clothing according to the sampler weight and size. The air adsorbing part of the sampler is set near the respiratory location, aiming to collect the inhaled portion of the air.

Table 2 summarized previous studies of PAH exposure measured by personal sampling. Tonne et al. [72] measured the 48-h exposure of 9 PAHs by 348 pregnant females via an individual sampler, and the results indicated a mean value of 7.966 ng/m^3^. Mu et al. [73] observed a 1.0 to 2.5 higher exposure level of most PAHs in the Wuhan participants than in the Zhuhai participants in a large community-based cohort. Duan et al. [74] collected individual exposure samples of PAHs for 126 volunteers in Taiyuan. They found that the exposure level of 15 PAHs for rural residents was lower than that of urban residents in the non-heating season (404 and 312 ng/m^3^, respectively) while comparable in the heating season (690 and 770 ng/m^3^, respectively) due to enhanced household cooking and heating. Han et al. [75] measured the personal exposure level of 12 PAHs for a panel of 80 elderly, and clarified that the 44.1% of induced cancer risk from PAH exposure resulted from gasoline vehicle emission. Smargiassi et al. [76] conducted repeated daily measurements of 72 asthmatic children living in an industry-surrounding region, with samplers loaded in a small backpack, and found comparable exposure concentrations of LMW-PAHs (2–4 ring PAHs) and HMW-PAHs (5–6 ring PAHs) with mean values of 145.38 and 150.66 μg/m^3^, respectively. Fan et al. [77] performed personal sampling on 46 volunteers living in Hong Kong and detected a contribution of 46.8 ± 13.4% of carcinogenic PAHs in the total 16 PAHs. Xu et al. [78] conducted personal sampling on subjects who were directly impacted by anthropogenic sources in an urbanizing region in southwest Africa and found that women’s (exposed to the emission of domestic cooking and heating, 77.4 ng/m^3^) exposure concentration to 19 PAHs was 1.6 and 2.1 times that of students (exposed to waste burning discharge, 49.9 ng/m^3^) and drivers (exposed to vehicle emission, 37.0 ng/m^3^), respectively. Wei et al. [79] measured the 24-h PAH exposure level on security guards, which was mainly generated from vehicular emission, and found it was related to increasing oxidative burdens. Lovett et al. [80] measured the exposure level in four commuter routes by letting researchers carry samplers during the transport period. They found that the highest PAHs along a freeway (0.940 ± 0.346 ng/m^3^) than a subway route, a light rail route, and high-density surface streets. Yan et al. [81] also detected the in-traffic exposure level by personal sampling and found that exposure in the bus mode (99.6 ng/m^3^) was higher than that in the walking mode (77.8 ng/m^3^) and subway mode (46.3 ng/m^3^).

Personal air monitoring is the most direct and accurate method for an external exposure measurement because it is not restricted to the spatial and temporal variations in atmospheric pollutants and personal activity. On the other hand, the accuracy of sampling is highly dependent on the participant’s ability to correctly carry and protect and record possible instrument failure. The high cost, time consumption, and participation burden restrict its application to investigations with a small sample size and a specific type of population (reducing generalization), or a short term and/or range.

### 2.3. Exposure Sampling Methods

The external exposure level can be measured by active or passive air samplers according to the research need. Active samplers, which rely on the power of a pump to collect pollutant-containing air, can be divided into real-time or offline analysis samplers. A real-time sampler can generate concentration data because of its quick response to the captured PAHs, which can record abrupt exposure events [82,83,84,85,86]. However, this technique has poor accuracy with regard to the identification of individual species [87,88]. Active samplers with offline analysis capabilities commonly collect airborne PAHs with filters or sorbent materials. Particulate phase PAHs are suitable for collection with quartz filters, Teflon filters, and glass fiber filters, while polyurethane foam (PUF) and other sorbent materials (e.g., XAD-2, XAD-4, Carbopack C, and Tenax) are commonly employed for gas-phase PAH capture [89]. After loading, PAHs are extracted from the collection materials and analyzed by techniques such as high-performance liquid chromatography and gas chromatography-mass spectrometry. Filter-based sampling can generate a comprehensive pollution profile with the characterization and specified quantification of individual and total PAHs, with multi-particulate sizes and particulate and gaseous phase distributions, as well as the simultaneous PM concentration and other PM-containing constituents if available [72,73,78,81]. Nevertheless, active samplers can burden users with pump noise and equipment weight.

Passive samplers typically contain an air receiving unit and a holder that collects the target constituents that rely on the molecular diffusion mechanism and the affinity of the absorbent to PAHs. Several adequate absorbent materials, such as silicone products and PUF and GC capillary integration units, were selected as passive samplers [90,91,92,93,94,95,96]. Silicone wristbands are light and pose no inconvenience to subjects who are in vulnerable or occupational groups, such as pregnant females and firefighters [93,95,96]. Passive samplers are inexpensive and user-friendly, and are more appropriate for gas-phase PAH sampling because particulate PAHs are not prone to diffuse into the gas phase due to a higher vapor pressure. Some researchers verified the sampling efficiency of passive sampling by the simultaneous active sampling data [97,98]. For example, Anderson et al. [98] tested the sampling performance of silicone wristband on 12 gas-phase PAHs by a simultaneous active sampling with a PUF cartridge (with the particulate PAHs collected by a quartz microfiber filter previously) in a personal sampling field work. They established a linear relationship between the PAH data captured by the wristband and PUF, indicating that air concentrations around the individual can be estimated by multiplying the partitioning coefficient with concentration data measured by the passive sampler. Nevertheless, research on the passive sampler capturing particulate PAHs is still lacking. Thus, prior to general application of the passive sampler, the collection performance, storage availability (PAH loss due to atmospheric reactions and molecular diffusion), and availability for chemical analysis (accurate extraction and purification) should be ensured in laboratory experiments and verified by comparison with active sampling in field studies.

### 2.4. Time-Activity Patterns

Time-activity patterns of the targeting population contain important exposure elements such as the time, location, and activity [99]. A questionnaire is a conventional and low-cost method with general applicability that can provide a detailed personal trajectory containing the location and duration related to exposure sources and activity during a sampling period. Additionally, a questionnaire can obtain a qualitative description of the exposure level through some targeted questions related to, for example, the cooking fuel, the cooking style, the number of cigarettes smoked, and the length of a smoking period [78,100]. However, this approach relies on participants that use field-dependent recall or hand-written diaries, which are completed every sampling day. Additionally, this method exhibits a memory bias, a high user burden, and a restriction in time resolution (usually 15–30 min). Some studies have utilized a Global Positioning System (GPS) system to mark dwelling environments by using a portable logger co-packed with samplers or a smart phone application, which can reduce the anthropogenic bias and improve data sensitivity [101,102]. This method can provide more detailed geological information but requires stable technological support and scientific time span division and integration with human activity.

**Table 1:** Previous studies of PAH exposure measured by stationary sampling.

Subjects	PAHs Phase and Number	Sampler Type	Site	Area
Older adults [61]	16 PAHs in PM_2.5_	Active sampler	Communities	Beijing, China
Commuter [62]	16 PAHs in dust	Direct collection	Bus stops	Qingyang, China
Residents [63]	18 PAHs in PM and gas phases	Active sampler	Ambient sites	Tarragona area, Europe
Residents [64]	28 PAHs in PM and gas phases	Active sampler	Indoor and outdoor of rural residences	Shandong, China
Residents [65]	28 PAHs in PM and gas phases	Active sampler	Indoor and outdoor of rural residences	Shanxi and Guizhou, China
Residents [66]	19 PAHs in PM	Active sampler	Indoor of residences	Xi’an, China
Residents [46]	16 PAHs in and gas phases	Active sampler	Indoor and outdoor of residences	Northern China
Office workers [67]	15 PAHs in PM_2.5_	Active sampler	Indoor and outdoor of an office	Jinan, China
Schoolchildren [68]	11 PAHs in PM_2.5_	Active sampler	Indoor and outdoor of a school	Beijing, China
Schoolchildren [69]	9 PAHs in PM_2.1_	Active sampler	Outdoor of two primary schools	Shanghai, China
Residents [70]	16 PAHs in PM_2.5_	Active sampler	Outdoor, office, residential home, dormitory	Beijing, China
Residents [71]	26 PAHs in PM_2.5_	Active sampler	Outdoor, residential indoor	Hong Kong, China
Residents [82]	24 PAHs in PM	Real- and non-real time active sampler	Campus	Mexico city, Mexico
Workers [83]	PAHs in PM	Real-time active sampler	Indoor of bars or restaurants	Santiago, Chile
Children [84]	PAHs in PM	Real-time active sampler	School bus	California, America
Residents [90]	9 PAHs	Passive samplers	Indoor and outdoor of residences	Central Appalachia, America
Pregnant women [92]	8 PAHs in the gas phase	Fan–Lioy passive sampler	Indoor and outdoor of residences	Detroit, America
Pregnant women [94]	8 PAHs in the gas phase	Fan–Lioy passive sampler	Outdoor of residences	California, America
Asthma patients [103]	16 PAHs in PM_2.5_	Active sampler	Ambient stations	Taiwan, China
Children [104]	4, 5, 6 rings PAHs in PM	Real- and non-real time active sampler	A center station and residences	California, America
Residents [105]	16 PAHs in PM_2.5_	Active sampler	Ambient stations	Taiwan, China
Young females [106]	9 PAHs in PM	Active sampler	Indoor and outdoor of residences	Kraków, Poland

**Table 2:** Previous studies of PAH exposure measured by personal sampling.

Subjects	PAHs Phase and Number	Sampler Type	Area
348 Pregnant females [72]	9 PAHs in PM_2_._5_	Active sampler	New York, America
224 Residents [73]	16 PAHs in PM_2_._5_	Active sampler	Wuhan and Zhuhai, China
126 Residents [74]	15 PAHs in PM and gas phases	Active sampler	Taiyuan, China
80 Elderly [75]	12 PAHs in PM_10_	Active sampler	Tianjin, China
72 Asthmatic children [76]	LMW- and HMW-PAHs	Active sampler	Montreal, Canada
46 Residents [77]	16 PAHs in PM_2_._5_	Active sampler	Hong Kong, China
6 Residents [78]	19 PAHs in PM_2_._5_	Active sampler	Southern West Africa
2 Security guards [79]	24 PAHs in PM_2_._5_	Active sampler	Beijing, China
Commuters [80]	9 PAHs in PM_2_._5_	Active sampler	California, America
Commuters [81]	19 PAHs in PM_2_._5_	Active sampler	Beijing, China
11 Firefighters [91]	32 PAHs	Active and passive sampler	Stockholm, Sweden
22 Pregnant females [93]	51 PAHs in passive sampler, 20 PAHs in active sampler	Passive sampler	New York, America
72 Firefighters [96]	14 PAHs	Passive sampler	Florida, America

### 2.5. Modeling Work

Modeling is a low labor-intensive and cost-less alternative for the exposure evaluation of a population on an individual, which can offset the spatial-temporal restrictions of environmental monitoring and the research burden of personal sampling. The procedure of the model is to screen out exposure-relevant variables by backward or forward regression methods and combine them with exposure data through an appropriate statistical relationship form. Aquilina et al. [107] compared the predictive ability of multiple statistical approaches, including a linear regression model, a time-activity weighted model, a univariate general linear model, and machine learning techniques, namely, decision trees and neural networks, to individual exposure. In addition, the predictable ability of the estimation models can be verified by several statistics parameters [108]. For example, the coefficient of determination can measure the variability caused by a dependent variable to the measured values; the index of agreement can estimate the variation of the predicted values around the observed mean; the root mean squared errors can calculate the total average difference between the measured versus modeled values; and, the Pearson’s coefficient can indicate the collinearity between the observed and predicted values.

Basic concentration data for exposure evaluation can be generated from outdoor monitoring, microenvironmental measuring, and personal sampling. Outdoor monitoring data are the most available and common data source for human exposure prediction [103]. The land-use model combines the land-use type, topographic parameters, and traffic features to generate regional air pollution levels. Noth et al. [104] performed a land-use regression to estimate the outdoor exposure level of 4–6 ring PAHs of children by the ambient level collected at a center station. The dependent variables were filtered based on the estimated effect and statistical significance of each parameter. The model fit was verified by the leave-one-out cross-validation, which measures the total average difference between the observed and predicted values. The final model revealed robustness and explained 81% of the between-house variability and 18% of the within-house variability based on the home outdoor level. A geological information system (GIS) is a typical spatial interpolation model that uses the concentration of known monitoring points to infer the concentration of unknown monitoring points, among which Kriging interpolation is the most representative spatial interpolation model. Lee et al. [105] predicted the individual exposure of PM_2.5_-bound PAHs by the simulated outdoor PAH level, which was refined to a high spatial resolution of 0.001 degrees with a GIS mapping approach. The estimated exposure data revealed a good correlation of 0.729 (*p* < 0.01) with that obtained from personal sampling. Some studies established exposure evaluation models based on microenvironmental and/or personal sampling work. Choi et al. [106] established an exposure predictive model for pregnant females based on personal, indoor, and outdoor monitoring data and found that indoor pollution closely reflects short-term exposure, while PAH exposure (Pyr and 8 carcinogenic PAHs) during the whole gestational period is more closely correlated with the outdoor pollution level. Wu et al. [101] successfully constructed regression models to estimate personal PAH exposures by coupling real-time PAH exposure, GPS-tracking time-activity data, and other exposure-related elements. The predicted data showed a correlation coefficient of 0.58 for daily exposures for all subjects, 0.61 for subject-dependent personal exposures, and 0.75 for subject-dependent exposures in different microenvironments. Based on a review of current studies, the successful modeling of outdoor exposure has been conducted, while studies of indoor environments still demonstrate a large gap. Although indoor environments contain contributions from indoor emissions and outdoor infiltration, current studies have mainly been focused on the outdoor contributions. The modeling of the indoor fraction has been limited because it ignores the diversity of the indoor microenvironments and simplifies the indoor impacting factors such as the deposition of pollutants, indoor air exchange, and building characteristics. Although many studies have explored the exposure features of microenvironments such as households, workplaces, and traffic, such results are restricted to similar exposure settings. Therefore, there is an urgent need to refine the research site and carry out general modeling work for a microenvironment exposure estimation.

## 3. Conclusions

This study summarized the evaluation methods for human inhalation exposure to PAHs, a group of toxic and ubiquitous compounds. External exposure utilizes environmental concentration coupling to describe human exposure, which can be measured under different spatial resolutions. Environmental monitoring focuses on ambient and other microenvironments by stationary samplers and can provide ample information on the exposure features of specific locations. Personal sampling is participant-based mobile monitoring, which is the most accurate approach to measure individual exposure without time-spatial restrictions. Human-exposed PAHs can be captured through an active sampler, which can provide a reliable and detailed description of PAHs in the gas and/or particulate phase, or passive samplers, which feature light weight, low user burden, and low cost while requiring qualified calibration. Time-activity patterns are a significant source of exposure parameters and can help to trace the exposure source and behavior and quantify the exposure level supported by time-activity diaries or GPSs.

Modeling work is generally applied to predict human exposure to PAHs by combining the exposure concentration with various exposure parameters by appropriate statistical approaches. It is significant to maintain a balance between the fitness and predictability power of the model. Furthermore, such work has successfully established ambient concentrations, while detailed modeling targeting various microenvironments, especially indoor microenvironments, is expected to be fulfilled to describe personal exposure more accurately.

## Data Availability

Data sharing not applicable.

## Acronyms and Abbreviations

5MC	5-methylchrysene
Ace	Acenaphthene
Acy	Acenaphthylene
Ant	Anthracene
BaA	benz[*a*]anthracene
BaP	benzo[*a*]pyrene
BbF	benzo[*b*]fluoranthene
BcF	benzo[*c*]fluorene
BghiP	benzo[*ghi*]perylene
BjF	benzo[*j*]fluoranthene
BkF	benzo[*k*]fluoranthene
Chr	Chrysene
CPP	cyclopenta[*c,d*]pyrene
DBA	dibenz[*a,h*]anthracene
DeP	dibenzo[*a,e*]pyrene
DhP	dibenzo [*a,h*] pyrene
DIP	dibenzo[*a,l*]pyrene
DiP	dibenzo[*a,i*]pyrene
Fle	fluorene
FR	fluoranthene
GIS	geological information system
GPS	global positioning system
HMW-PAHs	polycyclic aromatic hydrocarbons with low high molecular weight
IDP	indeno[1,2,3-*cd*]pyrene
LMW-PAHs	polycyclic aromatic hydrocarbons with low molecular weight
MMW-PAHs	polycyclic aromatic hydrocarbons with middle molecular weight
NaP	naphthalene
PAHs	polycyclic aromatic hydrocarbons
Phe	phenanthrene
PM	particulate matter
PUF	polyurethane foam
Pyr	pyrene
